# Cost-effectiveness analysis of long-course oxaliplatin and bolus of fluorouracil based preoperative chemoradiotherapy vs. 5x5Gy radiation plus FOLFOX4 for locally advanced resectable rectal cancer

**DOI:** 10.1186/s13014-019-1319-8

**Published:** 2019-06-24

**Authors:** Shichao Wang, Feng Wen, Pengfei Zhang, Xin Wang, Qiu Li

**Affiliations:** 1 Department of Radiation Oncology, Cancer Center and State Key Laboratory of Biotherapy, West China Hospital, Sichuan University, Chengdu, Sichuan Province People’s Republic of China; 2 Department of Abdominal Oncology, Cancer Center and State Key Laboratory of Biotherapy, West China Hospital, Sichuan University, 37# of Wainan Guoxue Lane, Chengdu, Sichuan Province 610041 People’s Republic of China; 3 West China Biostatistics and Cost-Benefit Analysis Center, Sichuan University, Chengdu, Sichuan Province 610041 People’s Republic of China; 4 Department of Medical Oncology, Cancer Center, State Key Laboratory of Biotherapy, West China Hospital, Sichuan University, 37# of Wainan Guoxue Lane, Chengdu, Sichuan Province 610041 People’s Republic of China

**Keywords:** Cost-effectiveness, Short-course radiotherapy, Long-course chemoradiotherapy, Rectal cancer, Hypofractional radiotherapy

## Abstract

**Purpose:**

To evaluate the cost-effectiveness of preoperative short-course radiotherapy (SCRT, 5 × 5 Gy) plus FOLFOX4 versus long-course oxaliplatin and bolus of fluorouracil based preoperative long-course chemoradiotherapy (LCCRT, 50.4 Gy in 28 fractions) in the management of cT4 or advanced cT3 rectal cancer (RC), both of which have been reported to achieve similar clinical effect in the NCT00833131 trial.

**Materials and methods:**

A Markov decision-analytic model compared SCRT plus chemotherapy and LCCRT, by simulating three health states (disease-free survival (DFS), progressive disease (PD) and death). The primary outcomes were quality-adjusted life months (QALMs), costs, and incremental cost-effectiveness ratios (ICERs). Transition probabilities were based on the NCT00833131 trial. The costs were calculated from a Chinese payers’ perspective. Strategies were evaluated with a willingness-to-pay (WTP) threshold of $2370.47 (3 × GDP) per QALM gained. Sensitivity analysis was performed to model uncertainty in these parameters.

**Results:**

The overall costs for SCRT plus chemotherapy and LCCRT were $78,937 and $38,140 with effectiveness of 29.92 QALMs and 22.99 QALMs, respectively. SCRT plus chemotherapy increased costs and QALM by $40,797.34 and 6.93 compared to LCCRT, resulting in an ICER of $5884.56/QALM gained. In the DFS state, the whole cost for SCRT plus chemotherapy and LCCRT were $11,490.03 and $10,794.06 with an effectiveness of 21.70 QALMs and 19.65 QALMs, respectively. SCRT plus chemotherapy increased cost and QALM by $695.97 and 2.05 compared to LCCRT, resulting in a ICER of $339.50/QALM gained, which below the WTP. The utility associated with the DFS state was the most influential factor on the cost-effectiveness of SCRT plus chemotherapy. When the cost of PD state below $1920, the ICER of SCRT compared with LCCRT below the WTP.

**Conclusion:**

Compared with LCCRT, SCRT plus chemotherapy is a more cost-effective strategy for locally advanced resectable RC in the DFS state as well as in the all states when the cost of PD state below $1920.

## Introduction

Rectal cancer (RC) is one of the most frequent malignancies in the world and represents a major socioeconomic and health issue [1]. In China, colorectal cancer (CRC) has attracted increasing attention over recent years, taking a second and fourth position in the incidence and mortality respectively among all malignant tumors in urban populations [2]. RC accounts for about 40% in the morbidity of CRC [2].

Surgery is the basic treatment for RC, but for advanced RC, adjuvant radiotherapy with or without chemotherapy has been used widely to improve outcomes. It is well known for locally advanced disease, postoperative chemoradiotherapy (CRT) significantly improves both local control and overall survival as compared with surgery alone or surgery plus irradiation [3, 4]. There are fewer studies on the preoperative treatments. Preoperative short-course radiotherapy (SCRT) plus chemotherapy consisting of FOLFOX4 or preoperative long-course chemoradiotherapy (LCCRT) with oxaliplatin and boluses of 5- fluorouracil and leucovorin in combination with conventional surgery are recommended depending on the tumor location, infiltration depth of the tumor, and lymph node involvement, which improves local control and survival [5]. Early results showed short-term preoperative radiotherapy decreased risk of local recurrence for irradiated patients at 2 years (2% vs 8%, *p* < 0.001) without a difference in overall survival (OS) for the patients with rectal cancer who undergo a standardized total mesorectal excision [6]. After a median follow-up of 6 years, the effect of radiotherapy on local recurrence persisted (6% vs 11%, *p* < 0.001), as well as the absence of a survival benefit for the patients with mobile rectal cancer undergoing surgery treated with preoperative short-term radiotherapy [7]. There is no international consensus on the use of these treatment schedules or the most appropriate patient selection for these schedules.

The phase III trial NCT00833131 comparing SCRT (5 × 5Gy) consisting of FOLFOX4 versus LCCRT (1.8 × 28Gy) in patients with fixed cT3 or cT4 rectal cancer with oxaliplatin and boluses of 5- fluorouracil and leucovorin, revealed that preoperative LCCRT, aimed at tumor shrinkage, is used to achieve R0 resection [8]. Nevertheless, improved OS and lower acute toxicity favours the 5 × 5Gy schedule with consolidation chemotherapy, with statistically-significant improvements found in disease-free survival (DFS) and OS with SCRT (*p* = 0.046) [8].

Several studies (e.g. CAO/ARO-094) have proved that preoperative radiotherapy has advantages in promoting the local control rates (LCR) and reducing toxicity compared with other treatment methods [9–13]. So far, no economic evaluation is available that compares preoperative SCRT with consolidation chemotherapy versus preoperative LCCRT. It is reported that SCRT with consolidation chemotherapy could be considered as an effective option for preoperative management in advanced rectal cancer, especially in countries with low health care budgets or long waiting lists for radiotherapy [8]. Given the increasing emphasis on value-based care in oncology, the aim of this study was to compare SCRT plus chemotherapy and LCCRT for the management of cT4 or advanced cT3 RC using a decision analysis approach.

## Materials and methods

### Decision model

A Markov model was used to estimate the overall benefits, risks and costs of SCRT plus chemotherapy compared to LCCRT by simulating a cohort of patients with rectal cancer over a 10 year time horizon. Model parameters were based on the literature and clinical data. TreeAge Pro 2016 (TreeAge Inc., Williamstown, Mass) was used for the Markov model.

**Fig. 1:**
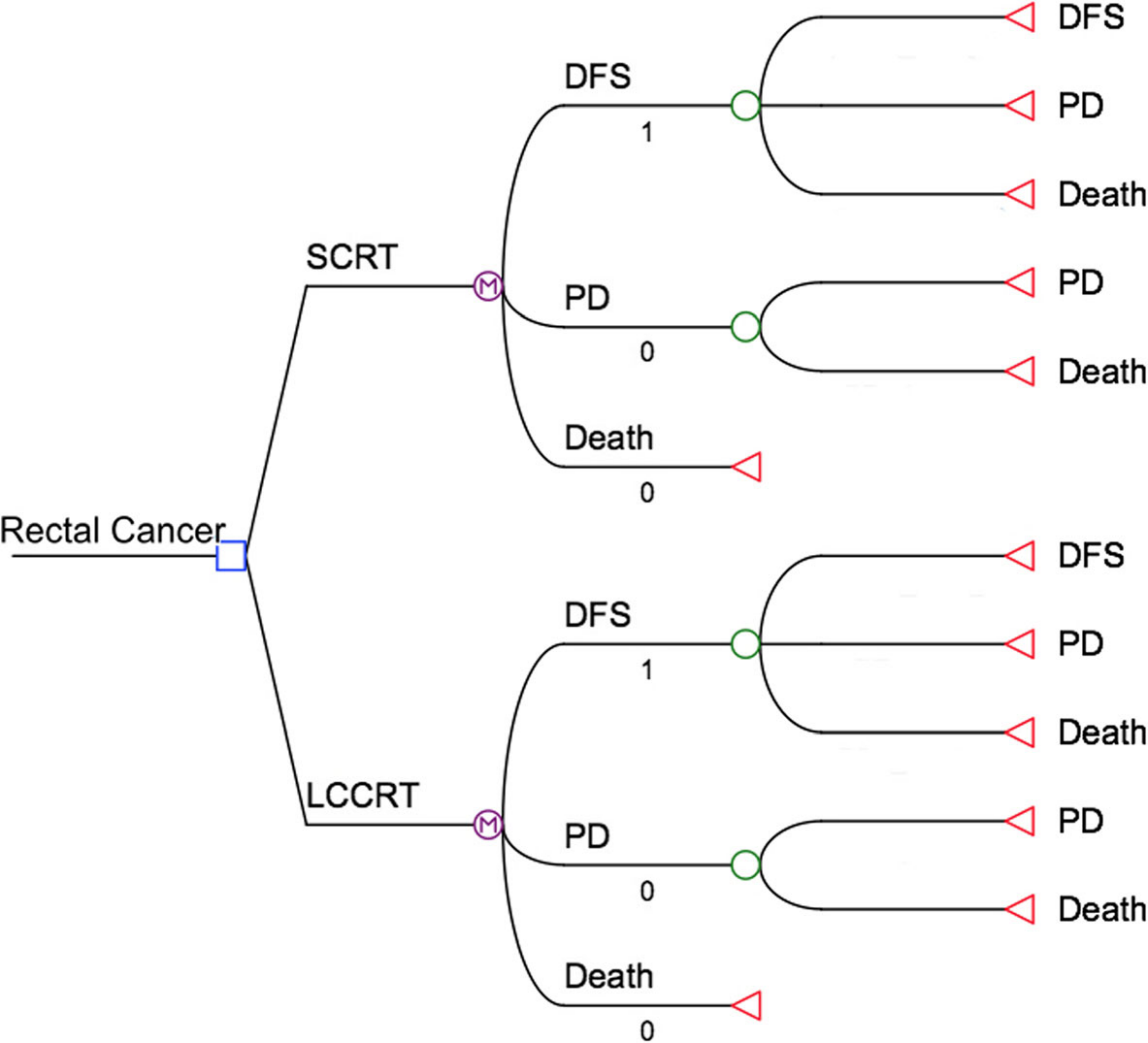
Markov model for advanced rectal cancer. According to the study profile, two groups were analyzed: group SCRT, patients with locally advanced resectable rectal cancer treated with preoperative short-course irradiation (5 × 5Gy) and immediate surgery plus chemotherapy; group LCCRT, patients with locally advanced resectable rectal cancer treated with long-course preoperative CRT (1.8 × 28Gy) and delayed surgery. A Markov model comprising three health states (disease-free survival (DFS), progressive disease (PD) and death) was built

In this study, we defined three health states: DFS, progressive disease (PD), and death (Fig. 1). A patient was considered to be in one of these health states at any given time. The Markov model was based on a payer’s perspective and ran with a 1-month cycle length.

All patients began in the DFS state after the CRT and surgery treatments, and had monthly potential transitions to PD or death. SCRT consisted with either preoperative 5 × 5Gy irradiation over 5 days with consolidation chemotherapy consisting 3 cycles of FOLFOX4. LCCRT consisted with preoperative LCCRT consisting 1.8 × 28Gy concomitantly with oxaliplatin and boluses of 5- fluorouracil and leucovorin. Surgery costs were also included in both arms of this study.

This is a retrospective study was approved by medical ethics committee of West China Hospital, Sichuan University, People’s Republic of China. None of the authors directly interacted with any study participants as the analysis was model-based and relied on inputs from the published literature and medical databases.

### Health state transitions

The monthly transition probabilities between the states were based on the Kaplan-Meier survival curves for DFS and OS for both strategies from the NCT00833131 trial [8]. To extrapolate the probability of survival during the observation period, Engauge Digitizer software was used to extract digitized data points from the Kaplan-Meier curves from the study. The Microsoft excel solver were used to calibrate 2-parametric Weibull survival models to the observed trial data by minimizing the sum of monthly squared deviations between the curves over a 6 year time horizon.

### Cost

Direct medical costs were considered as part of the payer perspective, which included costs of radiotherapy, chemotherapy drugs, surveillance (physician visit, blood tests, serum carcinoembryonic antigen (CEA) level test, computed tomography (CT) and colonoscopy), medical care (cost of care for metastatic CRC), adverse events. A detailed assessment of unit costs for these resources was conducted using data from the Cancer Center of West China Hospital of Sichuan University. All costs were converted into 2018 U.S. dollars based on the medical price index of National Health and Family Planning Commission of People’s Republic of China (PRC) and an exchange rate of $1 = ¥6.5 (Jun., 2018).

The total direct cost of radiotherapy was determined by multiplying resource use with the unit costs. Indirect department costs and overheads were allocated to treatments based on the number of fractions delivered. The total cost of radiotherapy was considered in the first cycle of the Markov model. The societal costs, such as travel costs and time costs, were not included in this study.

Because of limited information about the treatments for patients who were suffered from recurrence after the surgery in the NCT00833131 trial, the costs in the PD state were based on FIRE-3, a previously-published cost-effectiveness analysis from the Chinese perspective [14].

### Cost-effectiveness and utilities

The utility of the DFS and the PD state were based on published research. According to Ramsey’s study about colorectal cancer patients who received surgery [15], the utility of DFS was set at 0.84. The utility of the PD state was 0.6 based on Wen’s study [14]. Considering the treatment strategies of both groups were similar, the utilities for each state of both groups were assumed to be the same. The utilities were used to compute total quality-adjusted life months (QALMs) for each treatment [16]. The QALM is a combination of length of life and quality of life, with each month of life weighted by the utility that reflects the quality of life.

The incremental cost-effectiveness ratio (ICER) was calculated by dividing the difference in costs by the difference in QALMs. We considered both benefits and costs from a social perspective regardless of to whom they accrued and discounted both at a 3% annual rate. The cost-effectiveness analysis was carried out from a Chinese payer’s perspective. Model transition probabilities, costs, and utilities are included in Table 1.

**Table 1:** Transition probabilities, unit costs, and utilities used in the analysis

Parameter	SCRT	LCCRT	Data source
Survival			
3 year-DFS	53%	52%	Bujko et al. [8]
3-year OS	73%	65%	Bujko et al. [8]
Probabilities (monthly)			
DFS to PD	0.0204	0.0244	
DFS to Death	0.0148	0.0148	
PD to Death	0.0348	0.1020	
Costs per Cycle in US$			
DFS state	444.85	461.34	
PD state	4920.50	4920.50	Wen et al. [14]
Utility			
DFS state	0.65	0.56	Ramsey et al. [15]
PD state	0.47	0.47	Wen et al. [14]
Death state	0.00	0.00	

*Abbreviations: SCRT* Short-course radiotherapy, *LCCRT* Long-course chemoradiotherapy, *DFS* Disease-free survival, *OS* Overall survival, *PD* Progressive disease, *m* median

### Sensitivity analysis

We carried out a one-way sensitivity analysis to examine the influence of different parameters on the overall cost-effectiveness of treatment. The variables in the sensitivity analysis varied at a range of ±20%. In addition, to account for overall uncertainty we conducted a probabilistic sensitivity analysis were all parameters are varied simultaneously in a Monte Carlo simulation of 10,000 individuals and 1000 trials. Those results were reported as the percentage of trials in which a strategy is cost-effective at a series of willingness-to-pay (WTP) (or acceptability) thresholds in terms of dollars spent per QALM gained. We particularly focused on a threshold of 3 × the per capita GDP of China, which comports with the WHO guidelines for cost-effectiveness analyses [17]. Per capita disposable income of Chinese patients in 2017 was $790 per month, so the WTP threshold was $2370.47 (3 × GDP) per QALM.

## Results

### Patients characteristics

The Polish Colorectal Cancer Study Group enrolled patients in an open labeled, prospective, randomized phase III trial NCT00833131. Five hundred fifteen patients from 39 Polish institutions were randomly assigned either to SCRT plus chemotherapy or LCCRT. Two hundred sixty-one allocated to 5 × 5 Gy plus chemotherapy, 254 allocated to LCCRT among them. Based on the study, the 3 year disease-free survival and overall survival in SCRT plus chemotherapy and LCCRT were 53% vs. 52% (*p* = 0.85) and 73% vs. 65% (*p* = 0.046), respectively.

### Cost

SCRT plus chemotherapy cost much more than LCCRT ($78,937 vs. $38,140). Costs in the DFS state were slightly higher with SCRT plus chemotherapy than in LCCRT ($11,490 vs. $10,794) because of the slightly higher disease-free survival. Because overall survival was higher with SCRT plus chemotherapy, the costs accrued in the PD state were much higher with SCRT plus chemotherapy than LCCRT ($67,447 vs. $27,346). Overall, the incremental cost of SCRT plus chemotherapy over LCCRT was $40,797. Information regarding CRT and RT analyzed in the model is illustrated in Table 2. The costs of radiotherapy were considerably cheaper compared with chemotherapy agents in general.

**Table 2:** Cost for in US$ chemotherapy, hospitalization, test and surgery per patient

Cost item	SCRT	LCCRT
Radiotherapy		
Physician Consultation	14.7	14.7
CT Simulation	470 + 5.3	470 + 5.3
Physics planning	177	177
Treatment and positioning	3032	4928
Number of fractions delivered	5	28
Drug (Direct)		
Frequency	FOLFOX4	5-fluorouracil: 325 mg/m^2^/day leucovorin: 25 mg/m^2^/day
For the DFS state/m	39.31	38.16
For the PD state/m	79.33	57.95
Total cost	7064.01	5670.94
Hospitalization		
Caregiver (nurse)	52/day	52/day
Tests		
Surgery	2963.97	2645.13
Perioperative		
AE	6.12	5.49
Base+Follow Up	396.25 + 3357.58	330.04 + 3357.58

*Abbreviations: SCRT* Short-course radiotherapy, *LCCRT* Long-course chemoradiotherapy, *CT* Computed tomography, *PD* Progressive disease, *DFS* Disease-free survival, *AE* Adverse event

### Effectiveness

The total effectiveness was 29.92 QALMs for SCRT plus chemotherapy, and 22.99 QALMs for LCCRT. In detail, 21.27 QALMs were accrued in the DFS state and 8.22 QALMs in the PD state for SCRT plus chemotherapy. With LCCRT, 19.65 QALMs and 3.33 QALMs were accrued in DFS and PD states, respectively.

### Cost-effectiveness

**Fig. 2:**
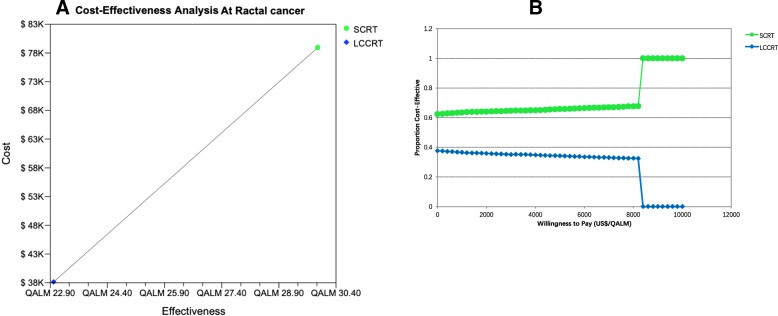
**a**: Cost-effectiveness pictured with two groups. Two groups were analyzed: group SCRT and group LCCRT patients with advanced resectable rectal cancer. **b**: Cost-effectiveness acceptability curves for SCRT and LCCRT strategies. Each curve shows the probability that the ICER for each treatment falls within a given willingness-to-pay threshold. QALM, Quality-adjusted life year; US$, United States dollars

Table 3 presented the results of the cost-effectiveness analysis. Incremental costs were $ 40797 and the incremental effectiveness was 6.93 QALMs in patients treated in SCRT plus chemotherapy compared with patients who received in LCCRT, resulting in an ICER of $5885/QALM gained (Fig. 2a).

**Table 3:** Results of cost-effectiveness analysis

Parameter	SCRT	LCCRT
Cost in US$		
Costs for the DFS state	14490	10794
Costs for the PD state	67447	27346
Total costs in US$	78937	38140
Incremental costs	40797	
Utilities		
Effectiveness (QALMs)		
Effectiveness for the DFS state	21.70	19.65
Effectiveness for the PD state	8.22	3.33
Total effectiveness	29.92	22.99
Incremental effectiveness	6.93	

*Abbreviations: SCRT* Short-course radiotherapy, *LCCRT* Long-course chemoradiotherapy, *PD* Progressive disease, *DFS* Disease-free survival, *QALM* Quality-adjusted life month

### Sensitivity analysis

**Fig. 3:**
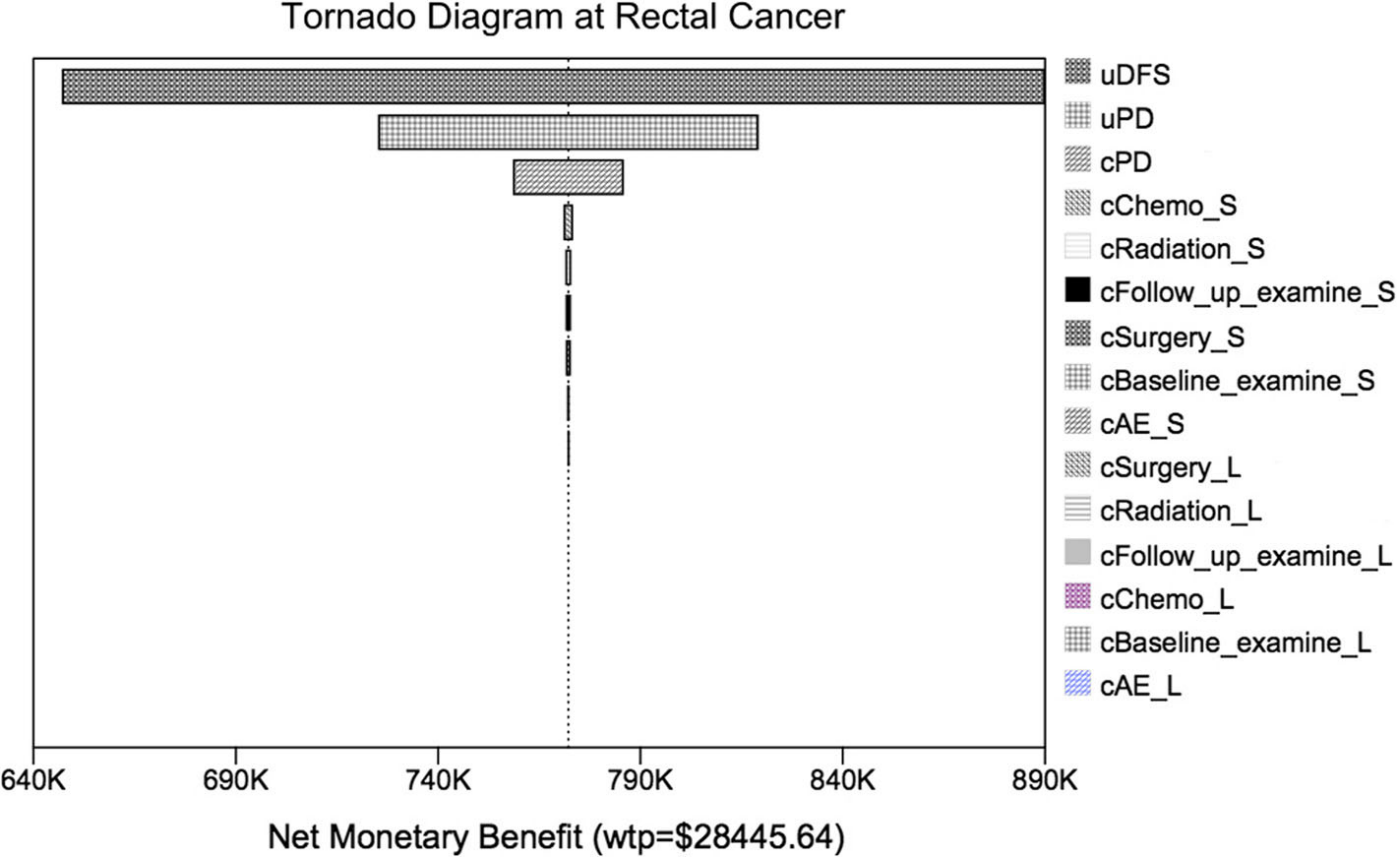
Tornado diagram of one-way sensitivity analysis. Tornado diagram summarized the results of one-way sensitivity analysis to identify model variables associated with the two strategies in the treatment of advanced gastric cancer

One-way and probabilistic sensitivity analyses were performed to test the robustness of the Markov model. The variables in the sensitivity analysis varied at a range of ±20%, and the results are shown as the tornado diagram in Fig. 3. Net monetary benefit (NMB) was applied in the tornado diagram. According to the calculation formula NMB = Effectiveness × WTP-Cost, NMB combines cost, effectiveness and WTP into a single measurement, and can show at what point in variable range do we have a change in the recommended strategy based on cost-effectiveness (Fig. 2b) . The WTP is set at $2370.47(3 × GDP) per QALM.

Results of the sensitivity analysis revealed that the utility of DFS state was the most influential factor for the robustness of the model, followed by transition probabilities in SCRT plus chemotherapy and LCCRT. On sensitivity analysis, as shown in Fig. 2a, there was a linear relationship between the feasibility rate of high-confidence differentiation between the two groups on the Chinese population, for testing the responsiveness of the model and the robustness of our results.

**Fig. 4:**
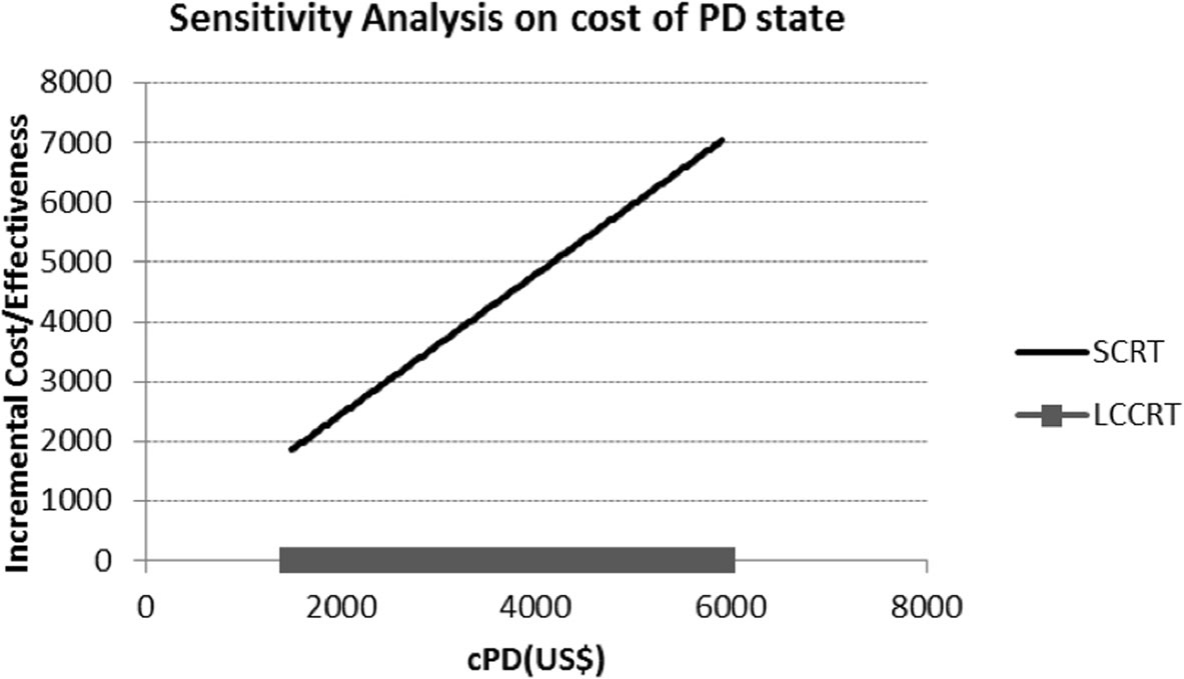
Sensitivity analysis on cost of PD state of two groups

Because the cost of PD state was borrowed from our previous study, it was a important factor affecting the results of our model. It was found that when the cost of PD state below $1920, the ICER of SCRT plus chemotherapy compared with LCCRT below the WTP (Fig. 4).

## Discussion

There have been plenty of studies published on the preoperational CRT regimens for RC which have been widely used in clinical practice. However, the research on economic analysis was rarely reported focusing the cost-effectiveness comparison. This is the first study to specifically investigate the cost-effectiveness between the preoperational SCRT plus chemotherapy and LCCRT for advanced RC patients.

One of the strengths of this study is that we included that preoperative SCRT plus chemotherapy consisting 3 cycles of FOLFOX4 may be a more cost-effective for locally advanced resectable RC, especially for the patients in the DFS state. The SCRT plus chemotherapy had more total cost than LCCRT with 5-fluorouracil and leucovorin, which maybe resulted from the long survival in PD state after the treatment of DFS state and the high cost of targeted therapies in PD state. But for the patients in DFS, the whole cost for SCRT plus chemotherapy and LCCRT were $11,490 and $10,794 with an effectiveness of 21.70 QALMs and 19.65 QALMs, respectively. In other words, SCRT plus chemotherapy increased cost and QALM by $695.97 and 2.05 relative to LCCRT, resulting in a ICER of $339.50/QALM gained, which below the WTP. Besides, our result was markedly consistent on deterministic and probabilistic sensitivity analyses, strengthening the argument that this novel technology is actually cost-effectiveness. The challenges facing healthcare worldwide is the incremental cost-effectiveness and the threshold for using or rejecting specific drugs. Based on the trial, SCRT with consolidation chemotherapy can be considered as an effective option for preoperative management in advanced RC, especially in countries with low health care budgets or long waiting lists for radiotherapy. Similar results were concluded by Mandy Van Den et al that short-term preoperative radiotherapy in patients with rectal cancer undergoing total mesorectal excision (TME) is both effective and cost-effective based on the trial conducted by the Dutch Colorectal Cancer Group [18]. And Dahlberg M et al found 5 × 5 Gy preoperative radiotherapy was in the range of other well-accepted medical interventions with reduced local recurrence rates and improved overall survival in a Swedish Rectal Cancer Trial [19].

On sensitivity analysis, the utility of DFS state was the most influential factor for the robustness of the model, followed by transition probabilities in SCRT plus chemotherapy and LCCRT. Especially, the costs in perioperative period had the greater impact on the results than the radiotherapy only. Furthermore, a key factor distinguishing between the two groups was the time arrangement for radiotherapy and chemotherapy, except for the surgery. Since SCRT plus chemotherapy costs much more than LCCRT ($78,937 vs. $38,140), cost for DFS state was higher in SCRT plus chemotherapy than in LCCRT, while cost for PD state was distinctly higher in SCRT plus chemotherapy than in LCCRT. There more chemotherapy drugs were given by infusion in LCCRT, which affected the stay-days in hospital. The costs of supportive drugs also showed a profound impact on the analysis model, which were related to patients’ length of stay. The longer the hospital stay, the more supportive drugs were administrated. It was suggested that the hospitals losses could be reduced by shortening the length of patients’ hospitalization. Dramatically, QALMs in the PD state for SCRT plus chemotherapy was distinctly higher than the data in LCCRT (8.22 vs. 3.33). There may be a balance between the cost and effectiveness after surgery. The results were consistent with Nishimura et al. [20], which suggested that the hospitals losses could be reduced by shortening the length of patients’ hospitalization, furthermore, increasing the dose per fraction is a good choice for the patient with RC.

Several limitations may not been ignored in this study. Principally, this study was based on a published clinical trial, rather than a randomized prospective research. It reflects the actual resource use of the subjects’ care but, of course, is subject to bias. Furthermore, the information about the PD state in the NCT00833131 trial was limited, the costs in this setting were based on the cost-effectiveness analysis from Chinese FIRE-3 study, which has been analyzed by our group previously. Data from many different sources could be reasonably combined into a single model to yield useful results, but this may have determined multiple biases in the analysis. For example, the adverse events maybe changed because of the different nationality in NCT00833131 trial and FIRE-3(Polish vs. Germany and Austria), leading to little cost discrepancy in the PD state. It’s worth noting that treatments to PD state is palliative care for advanced rectal cancer, so when the patients in the NCT00833131 trial translated to PD state, the strategies could be the same as patients in FIRE-3. At the same time, cost-effectiveness analysis for FIRE-3 study was from Chinese perspective, which was the same in the current study, so there was no nationality difference. Undeniably, although we assessed the economic costs in this study, it was reminded that some other factors biasing the analysis result would influence the choice of fit treatment protocol for the patients with RC, containing the time away from home, lost productivity costs, education, religion. Meanwhile, as Dahlberg et al concluded that the long-term side effects of radiotherapy and chemotherapy before surgery should be of future prime concern [19]. Based on these limitations, we still believe that our study will encourage decision makers to take a more comprehensive view of treatment- related costs.

## Conclusion

Compared to LCCRT, SCRT plus chemotherapy was more cost-effective for locally advanced resectable RC, despite the higher cost with SCRT plus chemotherapy has been shown in this study. Our analysis sharply suggested that it should be preferred to hypofractional radiotherapy in a cost-conscious environment for the patients in the DFS state as well as in the all states when the cost of PD state below $1920.

## Data Availability

The dataset generated and analyzed during the current study are available from the corresponding author on reasonable request.

## Abbreviations

CEA: Carcinoembryonic antigen
CRC: Colorectal cancer
CRT: Chemoradiotherapy
CT: Computer tomography
DFS: Disease-free survival
GDP: Gross domestic product
ICER: Incremental cost-effectiveness ratio
LCCRT: Long-course chemoradiotherapy
NMB: Net monetary benefit
OS: Overall survival
PD: Progressive disease
QALM: Quality-adjusted life month
RC: Rectal cancer
SCRT: Short-course radiotherapy
TME: Total mesorectal excision
WTP: Willingness-to-pay
